# Subjective strain of care experienced by pulmonary and critical care medical nurses when caring for patients with delirium: a cross-sectional study

**DOI:** 10.1186/s12913-021-06860-z

**Published:** 2021-08-12

**Authors:** Hongyi Tan, Lihua Zhou, Shuang Wu, Qiyu Dong, Liu Yang, Jiao Xu, Sue Zhao, Xiaoshan Wang, Hongzhong Yang

**Affiliations:** 1 Pulmonary and Critical Care Medicine Department, Huizhou Central People’s Hospital, Guangdong Medical University, No. 41, Eling Road, HuiCheng District, Guangdong 516001 Huizhou, People’s Republic of China; 2 Pulmonary and Critical Care Medicine Department, Changsha Central Hospital, University of South China, No. 161, Lushan South Road, Yuhua District, Hunan 410001 Changsha, People’s Republic of China

**Keywords:** Delirium, Nursing strain of care, Pulmonary care, Critical care nursing

## Abstract

**Background:**

Delirium, a disorder of consciousness, often occurs for a period of time during hospitalisation. It is characterised by a disturbance of attention or awareness. Hyperactive delirium may lead to accidental removal of medical equipment, while hypoactive delirium may inhibit patients from participating in nursing interventions, medical treatment, and physical therapy. However, there are limited relevant studies of the strain of care of nurses in China when caring for patients with delirium. This study, thus, aimed to investigate the subjective level of the strain of care experienced by pulmonary and critical care nurses when caring for patients with delirium.

**Methods:**

This was a descriptive, cross-sectional study. A survey was conducted with 100 nurses in the Chinese pulmonary and critical care medical (PCCM) department in 2018. The Strain of Care for Delirium Index (SCDI) was used to measure nurses’ strain of care. Participants were instructed to rate the degree of perceived difficulty in managing patients who displayed the behaviours listed in the SCDI, on a scale from 1 (quite easy) to 4 (very difficult). The mean ± standard deviation (SD) scores of the ranked difficulty scores were calculated.

**Results:**

In our sample, 47 % of the nurses had received delirium-related training previously. The three wards with the highest strain of care scores when caring for patients with delirium were the chronic obstructive pulmonary disease ward (3.29 ± 0.72), interstitial lung disease ward (3.11 ± 1.31), and respiratory intensive care unit (3.02 ± 0.78). The three types of patient behaviours associated with the highest degree of nursing strain of care were being uncooperative and difficult to manage (3.37 ± 0.84), pulling out tubes and tearing out dressings (3.33 ± 0.98), and irritability (3.22 ± 0.95).

**Conclusions:**

This study is the first to focus on nurses’ subjective strain of care when caring for patients with delirium in PCCM departments in China. The findings suggest the need to pay more attention to the working status of Chinese nurses. Further trials with large samples assessing relevant outcomes of patients with delirium are warranted.

**Supplementary Information:**

The online version contains supplementary material available at 10.1186/s12913-021-06860-z.

## Background

Delirium, a disorder of consciousness [1], may be attributable to substance withdrawal, medication, physiological consequences of another medical condition, or multiple other aetiologies. One essential feature is the disturbance of attention or awareness, accompanied by a change in baseline cognition that cannot attributed to a pre-existing or evolving neurocognitive disorder (NCD) [2]. Delirium is strongly associated with multiple adverse outcomes, such as falls, aspiration pneumonia, distress, and other events occurring during delirium or in the days following an episode [3]. The development and duration increases the risk of cognitive, psychiatric, and physical impairments, which accumulates over time and reduces a patient’s quality of life. Patients with delirium have higher mortality rates, longer mechanical ventilation time, longer hospital stays, and a higher risk of cognitive impairment [4–6]. A study found that the 30-day and 90-day mortality rate of patients in an intensive care unit (ICU) was 27 and 34 %, respectively [7].

Delirium is very common among older inpatients. Among those aged 70 years and older, more than one-third develop delirium during admission or hospitalisation [8]. Previous studies showed that delirium occurred in more than 75 % of patients who received mechanical ventilation in ICUs and could occur in up to 85 % of end-stage patients [7–9]. In pulmonary and critical care medical (PCCM) departments, most patients are older adults with chronic diseases, and some require mechanical ventilation or sedation. Our PCCM nurses also face the problem of delirium, especially in the ICU. In clinical practice, nurses in our healthcare facility use CAM-ICU or 3D-CAM to identify patients with delirium and provide multiple interventions, such as environment control and early pulmonary rehabilitation, to improve patient outcomes.

Several researchers have reported that behaviours associated with delirium hindered the provision of medical care. Hyperactive delirium leads to accidental removal of equipment, whereas hypoactive delirium inhibits patient participation in nursing interventions, medical treatment, and physical therapy [10, 11]. The occurrence of delirium in patients can lead to increased work burden on nurses, as providing care for such patients is extremely challenging and stressful [12]. Nurses often feel uncomfortable and unknowledgeable when caring for these patients [13]. However, to the best of our knowledge, no relevant studies have examined the strain of care of nurses in China when caring for patients with delirium. Thus, this study aimed to empirically investigate the subjective strain of care of nurses who cared for delirium patients and provide data for improving nursing work arrangements and enhancing nursing care.

## Methods

### Design

This study used a cross-sectional, descriptive survey design.

### Setting and sample

The PCCM department of Changsha Central Hospital in Hunan, China, includes six subspecialised wards for infectious lung disease, chronic obstructive pulmonary disease, interstitial lung disease, lung cancer, pulmonary rehabilitation, and a respiratory intensive care unit (RICU). There are 118 licensed nurses working in the PCCM department. A convenience sample of registered nurses was recruited for this study. The inclusion criteria were nurses who were present at the department during the morning shift when the questionnaire was administered. On the day of the survey, 100 nurses were included in the study. Eighteen nurses who were off-duty or unemployed were excluded.

### Data collection

As shown in Table 1, the Strain of Care for Delirium Index (SCDI) was used to assess participants’ strain of care in the present study. Milisen et al. [14] established the content and construct validity of the original instrument. To facilitate participants’ understanding, the questions were translated into Chinese by the researchers. The questions were slightly revised in keeping with China’s specific cultural and social contexts. A back translation was performed, and the results were shown to be the same as the original scale.

**Table 1:** Nurses’ strain of care when caring for patients with different symptoms of delirium

		Very easy	Easy	Difficult	Very difficult
Hypoactive behaviour	1. Avoidance and abnormal quietness	1	2	3	4
	2. Mental depression and lack of motivation	1	2	3	4
	3. Reduced activities	1	2	3	4
Mixed level of activity	4. Lack of awareness or understanding of own situation or disease	1	2	3	4
	5. Difficulty concentrating and easily distracted	1	2	3	4
	6. Speaking slowly or indecisively	1	2	3	4
	7. Avoiding eye contact	1	2	3	4
	8. Recalling incorrect names of familiar people	1	2	3	4
	9. Talking to someone who is actually not present	1	2	3	4
	10. Repetitive behaviour	1	2	3	4
	11. Speaking incoherently	1	2	3	4
	12. Alternating between clarity and confusion	1	2	3	4
Hyperactive behaviour	13. Sleep-arousal cycle disorder	1	2	3	4
	14. Irritability and excruciating	1	2	3	4
	15. Shouting loudly	1	2	3	4
	16. Irritability	1	2	3	4
	17. Increased activity	1	2	3	4
	18. Uncooperative and difficult to manage	1	2	3	4
	19. Unplanned attempts to get out of bed	1	2	3	4
	20. Pulling out tubes and tearing out dressings	1	2	3	4

The SCDI questionnaire comprises four sections, including a total of 20 questions related to nurses’ experience in managing the behaviours of patients with delirium. The questionnaire was distributed to and collected from nurses across all six wards on the same day. During the morning shift, approximately 10–15 min was used to communicate the instructions on completing the SCDI to the nurses in each ward. Participants completed the questionnaires independently. To prevent other participants from becoming knowledgeable about the content of the SCDI prior to participation, all participants were required to complete the SCDI on the same day.

Basic demographic data were obtained, and participants were instructed to rate the degree of perceived difficulty in dealing with patients who displayed the behaviours listed in the SCDI. Participants rated all items on a scale ranging from 1 (quite easy) to 4 (very difficult). The survey took approximately 20 min. For additional confirmation, data were collected again from the same 24 RICU participants two weeks later, resulting in a correlation coefficient of 0.90, which confirmed the test-retest reliability of the measure.

### Ethical considerations

 This study was approved by the Ethics Committee of the Changsha Central Hospital, University of South China. The study followed the ethical standards outlined in the Declaration of Helsinki. All participants were recruited by the head nurse after the shift meeting, and participation was voluntary. Before entering the study, all potential participants carefully read and signed an informed consent form.

### Statistical analysis

We analysed the data using GraphPad Prism 7 software. In the demographic information table, frequency count data were calculated for the percentage composition. The mean ± standard deviation (SD) of the ranked difficulty scores, which ranged from 1 to 4, collected from different wards, were calculated and compared using a one-way analysis of variance.

## Results

Of the surveyed nurses, 97 % were female, and most participants were young (aged 20–29; 69 %) (Table 2). Regarding education level, 58 % of nurses had completed technical secondary school, 26 % completed junior college, and 16 % had a bachelor’s degree. The length of service of 54 % of the participants was 2–5 years. Further, 24 nurses worked in the RICU, 20 in the infectious lung disease ward, 15 in the chronic obstructive pulmonary disease ward, 15 in the interstitial lung disease ward, 13 in the lung cancer ward, and 13 in the pulmonary rehabilitation ward (Table 3). Overall, 47 % of the nurses stated that they had received delirium-related training, primarily in-hospital training (44 %). The remaining 3 % stated that they had acquired relevant knowledge through self-training-related tutorials or by reading relevant literature. None of the nurses obtained relevant knowledge as part of their higher education. Notably, all 24 RICU nurses had received delirium-related training. All nurses worked 8 h a day on average; the main difference between the nurses working in the normal ward and those in the ICU was the number of patients they were caring for (Table 2).

**Table 2:** Demographic characteristics of the participants (*N* = 100)

Variable	*n*	%
Gender		
Male	3	3
Female	97	97
Age range (years)		
20–29	69	69
30–39	29	29
40–49	2	2
Education level		
Technical secondary school	58	58
Junior college	26	26
Undergraduate	16	16
Length of service (years)		
< 1	7	7
2–5	54	54
6–10	23	23
> 10	16	16
Received training about delirium?		
Yes	47	47
No	53	53
Method of training		
In-school training	0	0
Continuing education	47	47
In-hospital training	44	44
Self-training	3	3
Workload		
Caring for 3–4 patients in the RICU, 8 h/d	24	24
Caring for 12–15 patients in a normal ward, 8 h/d	76	76

**Table 3:** Average difficulty score of nurses when caring for patients with delirium in different wards

Ward (number of nurses)	Difficulty score	Rank
Infectious lung disease (20)	2.76 ± 0.90	4
Chronic obstructive pulmonary disease (15)	3.29 ± 0.72	1
Interstitial lung disease (15)	3.11 ± 1.31	2
Lung cancer (13)	2.38 ± 0.85	6
Pulmonary rehabilitation (13)	2.59 ± 0.85	5
RICU (24)	3.02 ± 0.78	3

* F*(5, 94) = 2.028, *p =* 0.082

A comparison of the means of the SCDI score of each ward showed that the top three wards where nurses experienced the highest subjective pressure when caring for patients with delirium were the chronic obstructive pulmonary disease ward (3.29 ± 0.72), interstitial lung disease ward (3.11 ± 1.31), and RICU (3.02 ± 0.78). These were followed by the infectious lung disease ward (2.76 ± 0.90), pulmonary rehabilitation ward (2.59 ± 0.85), and lung cancer ward (2.38 ± 0.85). However, there were no significant differences in the subjective strain of care scores between the wards (*F*(5,94) = 2.028, *p =* 0.082).

The mean difficulty score of the entire sample was 2.94 ± 1.77. Items 1 (avoidance and abnormal quietness, 2.38 ± 0.71), 2 (mental depression and lack of motivation, 2.38 ± 0.65), and 3 (reduced activities, 2.48 ± 0.66) were rated as the least challenging behaviours. Meanwhile, items 18 (uncooperative and difficult to manage, 3.37 ± 0.84), 20 (pulling out tubes and tearing out dressings, 3.33 ± 0.98), and 16 (irritability, 3.22 ± 0.95) were rated as the most challenging behaviours (Table 4). Item 18 (uncooperative and difficult to manage) was consistently ranked among the top three most difficult behaviours in all wards, and item 20 (pulling out tubes and tearing out dressings) was considered the most difficult to manage by nurses in the chronic obstructive pulmonary disease ward, interstitial lung disease ward, and RICU.

**Table 4:** Difficulty scores and index ranks for nurses’ strain of care when caring for patients with delirium

Rank	Difficulty score	Delirium behaviours
1	3.37 ± 0.84	18. Uncooperative and difficult to manage
2	3.33 ± 0.98	20. Pulling out tubes and tearing out dressings
3	3.22 ± 0.95	16. Irritability
4	3.20 ± 0.96	15. Shouting loudly
5	3.16 ± 0.88	19. Unplanned attempts to get out of bed
6	3.14 ± 0.95	14. Irritability and excruciating
7	3.11 ± 0.76	17. Increased activity
8	2.98 ± 0.83	12. Alternating between clarity and confusion
9	2.86 ± 0.74	13. Sleep-arousal cycle disorder
10	2.85 ± 0.77	9. Talking to someone who is not actually present
11	2.82 ± 0.88	11. Speaking incoherently
12	2.82 ± 0.74	10. Repetitive behaviour
13	2.79 ± 0.64	5. Difficulty concentrating and easily distracted
14	2.77 ± 0.69	4. Lacking awareness or understanding of own situation or disease
15	2.66 ± 0.73	8. Recalling incorrect names of familiar people
16	2.61 ± 0.63	7. Avoiding eye contact
17	2.58 ± 0.70	6. Speaking slowly or indecisively
18	2.48 ± 0.66	3. Reduced activities
19	2.38 ± 0.65	2. Mental depression and lack of motivation
20	2.38 ± 0.71	1. Avoidance and abnormal quietness
Average	2.94 ± 1.77	Entire sample

In all wards, the subjective strain of care for managing avoidance and abnormal quietness was the lowest, and this behaviour had the highest frequency of occurrence (four times). Mental depression and lack of motivation, reduced activities, and lack of awareness or understanding of one’s own situation or disease were all ranked second, with three occurrences each.

## Discussion

The SCDI [14] was used to investigate nurses’ difficulties in managing the individual aspects of delirium, and the subscales helped identify the types of delirium considered most difficult to manage. The mean subjective strain of care score of participants in this study was high (2.94 ± 1.77). This is similar to the score (M = 2.97) reported in a study by McDonnell and Timmins [12] and higher than that (M = 2.55) reported by Detroyer et al. [15]. The reason for this difference may be related to the participants’ different areas of work. In this study and the study by McDonnell and Timmins, most participants worked in an ICU, RICU, CCU, or emergency ward, or cared for older patients. Meanwhile, 59 participants in Detroyer et al.’s study worked in 20 inpatient units; this is in stark contrast to the present study, which recruited participants from the PCCM department alone. Primarily, hyperactive delirium increases nurses’ subjective strain of care [16]. Patients who were uncooperative and difficult to manage caused the highest degree of subjective strain for participants in this study, and these patients were deemed the most challenging to manage. However, most delirium patients experience hypoactive or mixed levels of activity. Patients who are quiet and have significantly reduced activity cause the lowest subjective strain of care for nurses; the presence of delirium in such patients is the most difficult to identify, is rarely noticed, and has a poorer prognosis [17].

Although there were no significant differences in the stress index between nurses from different wards, the nursing strain of care in the chronic obstructive pulmonary disease ward, interstitial lung disease ward, and RICU were higher than those of the other wards, which may be related to the fact that older patients in these wards usually have respiratory failure and may be in a critical condition. Respiratory failure [18], advanced age [8], and mechanical ventilation [9]—all of which are common in the PCCM department—are important risk factors for delirium in patients. In addition, glucocorticoids, which are commonly used in the PCCM department, are also an important factor leading to the occurrence of delirium [19]. Under the same nurse/bed ratio, the nurses in these three wards may have had to care for more delirium patients, thereby experiencing a higher degree of subjective strain of care. With respect to the mean difficulty scores of nurses in different wards, RICU nurses ranked third, which was surprising. It may be that RICU nurses have appropriate knowledge of delirium, can identify the situation early on, and obtain support from doctors to provide pharmacological or non-pharmacological interventions [12].

The participants in this study were young, and the proportion of nurses who had received delirium education was higher than that in a previous study [10], considering that all 24 nurses in the RICU had received delirium-related training. Early detection of delirium is key to managing delirium effectively; care providers providing timely treatment can improve patient prognosis [20]. Long-term investment in delirium-related training for nurses is necessary [21]. However, all nurses who had received delirium-related education denied having mastered delirium-related knowledge through school courses, and the overall proportion of nurses who received relevant training was low. Therefore, there is a need for more relevant delirium-related training in nursing education and work, and more e-learning tools [15] in China.

### Limitations

This study has a few limitations. First, only one instrument was used to assess nurses’ subjective strain of care. The problem of self-reported bias is also relevant with regards to the scale used. Self-report studies have many advantages; however, one of the disadvantages is that self-reported answers may be exaggerated or under-reported, depending on participants’ motivation. Future studies should target a more diverse demographic, including older nurses, and employ additional measures to assess their strain of care. Second, the scope of the present study was limited, in that it did not comprehensively examine the relationship of strain of care with contextual variables, such as patient characteristics, nursing context, and the extent of training, which may also influence nurses’ subjective strain of care. Thus, future studies should address this gap by examining these factors in relation to nurses’ strain of care. Third, this study was conducted under the premise of a good medical-nursing relationship. All nurses actively participated, but it was impossible to evaluate whether the working relationship between the investigator and participants affected the results of this study. This study was conducted by doctors and two head nurses. The 2 head nurses participated in the scale design, recruitment, as well as data collection throughout the entire process. Further, the data analysis and discussion were mainly completed by the participating doctors, which may have affected the discussion. Finally, the SCDI has rarely been used in research, and some of its content is outdated. Although we made some modifications to the items, the content of the 20 questions remained unchanged, so it is unlikely that the results were affected.

## Conclusions

To the best of our knowledge, this study is the first to focus on the subjective strain of care of nurses caring for patients with delirium in PCCM departments in China. Primarily, nurses working in the PCCM department reported a greater degree of strain of care from patients with hyperactive delirium symptoms compared to hypoactive patients. Nursing education in China provides very little training in delirium management; this warrants further improvement of nursing education to provide more hands-on, real world training to manage delirium.

## Supplementary Information

**Additional file 1.**

**Additional file 2.**

## Data Availability

All data generated or analysed during this study are included in this published article [and its supplementary information files].

## Abbreviations

ICU: Intensive care unit
PCCM: Pulmonary and Critical Care Medical
RICU: Respiratory intensive care unit
SCDI: Strain of Care for Delirium Index
